# The deer play in Wuqinxi and four-point hand−knee kneeling positions for training core muscle function and spinal mobility

**DOI:** 10.3389/fbioe.2022.965295

**Published:** 2022-09-27

**Authors:** Xiao-Qian Chang, Xin-Peng Chen, Yi-Xin Shen, Kuan Wang, Shang-Jun Huang, Yan Qi, Wen-Xin Niu

**Affiliations:** ^1^ Shanghai YangZhi Rehabilitation Hospital (Shanghai Sunshine Rehabilitation Center), School of Medicine, Tongji University, Shanghai, China; ^2^ Laboratory of Biomechanics and Rehabilitation Engineering, School of Medicine, Tongji University, Shanghai, China

**Keywords:** Wuqinxi exercise, core stabilization exercise, surface electromyography (EMG), vertebral rotation, core muscle stability

## Abstract

The four-point kneeling exercise is a core stabilization exercise that provides the spine with dynamic stability and neuromuscular control. In the traditional Chinese exercise Wuqinxi, deer play is performed in a hand−foot kneeling (HFK) position, which is remarkably similar to the four-point hand−knee kneeling (HKK) position. However, the differences in spinal function promotion between these two positions are poorly understood. The aim of this study was to investigate muscle activation patterns and spinal kinematics during specific core stabilization training to provide evidence for selecting specific exercises. A total of 19 healthy adults were recruited to perform HFK and HKK. The rotation angle of the C7–T4 vertebra and the surface EMG signals of abdominal and lumbar muscles on both sides were collected. The paired *t*-test showed that the vertebral rotation angles were significantly higher during HKK than HFK, and the intra-group differences mainly occurred at the level of the thoracic vertebra. The muscle activation of both sides of the rectus abdominis and external oblique in HFK was significantly higher than in HKK when the upper limb was lifted (*p* < 0.05). The activation of the ipsilateral lumbar multifidus and erector spinae muscles was significantly higher during the HKK position than during HFK when the lower limb was lifted (*p* < 0.05). HFK provided more training for strengthening abdominal muscles, while HKK could be recommended for strengthening lumbar muscles and increasing spine mobility. These findings can be used to help physiotherapists, fitness coaches, and others to select specific core exercises and develop individualized training programs.

## Introduction

Core stabilization exercise (CSE) is designed to promote the muscular co-activation patterns and the stability of the spinal structures (Essendrop et al., 2002; Brown and McGill, 2008; Oliva-Lozano and Muyor, 2020). It has been widely used in training programs to improve health and physical fitness as well as in clinical rehabilitation for the elderly (Areeudomwong and Buttagat, 2019; Ozsoy et al., 2019; Kim et al., 2020; Vera-Garcia et al., 2020). A well-trained core is essential for optimal performance and injury prevention (Cortell-Tormo et al., 2017).

Four-point kneeling exercise is regarded as a CSE that provides dynamic stability and neuromuscular control to the spine (O'Sullivan, 2000; Martuscello et al., 2013; Cortell-Tormo et al., 2017). It is a quadruped position, with the hip and knee at 90° flexion while maintaining abdominal hollowing (Stevens et al., 2007b; Pirouzi et al., 2013). To distinguish it from the other positions in this article, we call it the four-point hand−knee kneeling (HKK) position. Wuqinxi is a traditional Chinese fitness exercise (Huang, 2020) that is a set of exercises based on the typical movements of five animals: the tiger, deer, bear, ape, and bird (Guo et al., 2018). The positions between the five movements are quite different, and each movement is designed to promote a particular body function (Li et al., 2020; Chang et al., 2022). The deer play in Wuqinxi is played in a hand−foot kneeling (HFK) position, which is remarkably similar to the conventional core stabilization exercise, the four-point hand−knee kneeling (HKK) position. These quadruped positions provide relatively low-loaded and non-anti-gravity postures and could be appropriate choices for people with low back pain or spinal disorders starting a rehabilitation program.

Previous studies have shown that HKK and its variants could activate the abdominal and lumbar muscles, thereby enhancing lumbar spine stability (Sakulsriprasert et al., 2015). In the starting position, the electromyography (EMG) amplitudes of abdominal and lumbar muscles were generally lower than 20% (Chanthapetch et al., 2009; Park and Lee, 2010). HKK with the upper limb lifted had higher ipsilateral internal oblique and transversus abdominis muscle activation (Pirouzi et al., 2013). Another study found that the lower limb extension task in four-point kneeling provides both low joint loading and limited muscle activity, and this result suggested that changes in the limb position could have an effect on the core muscle activity pattern (Yu et al., 2022). Compared to HKK, the most distinctive feature of HFK is that the knee does not touch the ground. However, the differences in core muscle activity caused by the positions are not clear until now.

Spinal intervertebral joints are complex structures allowing motion in flexion−extension, lateral bending, and axial rotation. Spinal rotation has been proposed as a necessary motion for our everyday activities (Gombatto et al., 2015; Reitmaier and Schmidt, 2020). A previous study showed that an artificial restriction of spinal rotation resulted in significantly slower walking velocity and higher energy consumption in walking (Kumar, 2004). Moreover, both abdominal and dorsal muscles are involved in the development of axial torque (McGill. 1991). Kumar et al. (2002) also reported that the pre-rotated spine decreased force production significantly and increased EMG activity significantly. In the current study, we also measured the two quadruped positions with different limb extensions, which were unstable positions with asymmetrical body postures. Measuring spinal rotation is important for the evaluation of certain tasks involving asymmetrical body posture, which could provide not only information related to the rotation angles but also information about muscle activity patterns (Kouwenhoven et al., 2006; Fan et al., 2014).

Therefore, the purpose of this research was to investigate trunk muscle activation and spine kinematics to compare the differences between these two quadruped positions in training core muscle function and spinal mobility. It was hypothesized that physical demands differ between these two quadruped positions, which might be used in training for different spinal disorders. In this way, a normative database can be created, which is necessary to interpret the results of performing these exercises.

## Materials and methods

### Participants

A total of 19 healthy university students (12 males and 7 females) were recruited in this study according to the paired *t*-test, with a power of 0.80, an alpha level of 0.05, and an effect size of 0.68 (Kim, 2015). The sample size was calculated using G*Power software (v3.1.9.2) based on the pre-experiment in which the activation of the rectus abdominis was compared between these two different four-point kneeling exercises.

Their age was 21.8 ± 0.9 years, height was 169.9 ± 9.5 cm, weight was 61.9 ± 10.2 kg, and body mass index (BMI) was 21.3 ± 2.0 kg/m^2^. All participants were free from musculoskeletal pain, neuromuscular disorders, or any form of joint or bone disease. Participants with BMI greater than 28 kg/m^2^ were also excluded, aimed to decrease the EMG artifact due to adipose tissue lying between surface electrodes and tested muscles. All participants were informed about the purpose and content of the investigation and signed informed consent. Written informed consent was obtained from the individuals for the publication of any potentially identifiable images or data included in this article.

### Experimental design

All participants performed HKK and HFK and their variants in random order. Each movement was performed 2 times and held for 10 s. Rest periods of 60 s were allowed between repetitions of the movements, and a 2-min rest period was given between movements minimizing the possibility of residual fatigue (Bjerkefors et al., 2010a). Each participant was trained to perform movements in two positions, and this assured that they could correctly perform the movements. The two exercises and their variants were briefly described in the following paragraphs.

**FIGURE 1:**
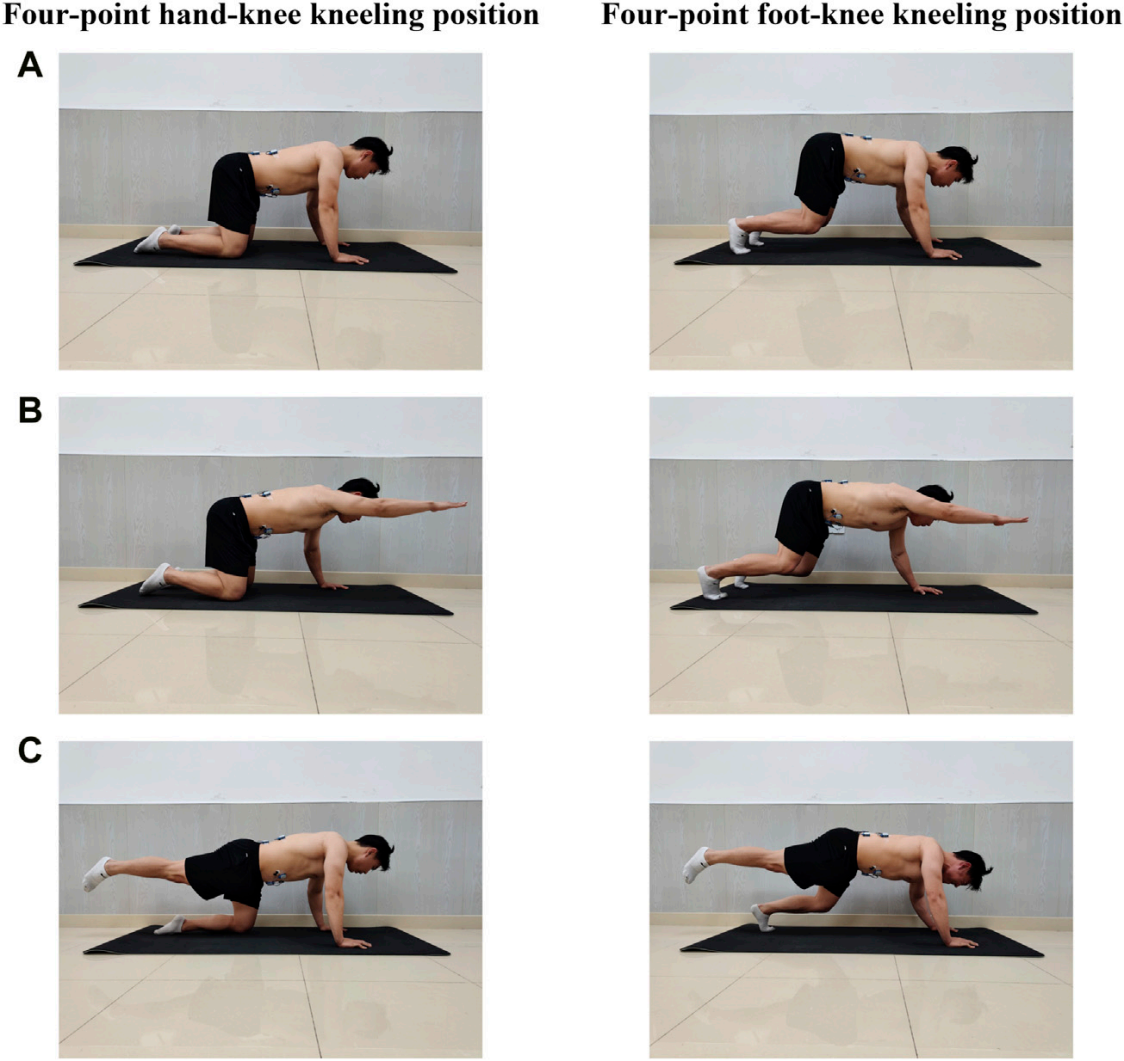
Core stabilization exercises in two positions. **(A)** Exercises in the starting position. **(B)** Exercisers with a single upper limb lifted; participants lifted their right or left upper limb parallel to the floor, respectively. **(C)** Exercisers with a single lower limb lifted; participants lifted their right or left lower limb parallel to the floor as possible, respectively.

HKK in the starting position: participants were in the quadruped position with hips and shoulders flexion at approximately 90°. Their hips were directly above the knees, and their shoulders were above the hands. The spine was in a neutral position (Figure 1A). HKK with the right or left upper limb lifted: from the starting position, participants lifted their right or left upper limb parallel to the floor, respectively (Figure 1B). HKK with the right or left lower limb lifted: from the starting position, participants lifted their right or left lower limb parallel to the floor, respectively (Figure 1C).

HFK in the starting position: participants were in the quadruped position with hips, knees, and shoulders flexion > 90°, the knee joints did not touch the ground, and the spine was required to be in a neutral position as possible (Figure 1A). HFK with the right or left upper limb lifted: from the starting position, participants tried to lift the right or left upper limb parallel to the floor, respectively (Figure 1B). HFK with the right or left lower limb lifted: from the starting position, participants lifted their right or left lower limb as parallel to the floor as possible, respectively (Figure 1C).

### Surface electromyography data acquisition and processing

The surface electromyography (sEMG) signal acquisition was performed using Noraxon TeleMyo DTS (Noraxon Inc., AZ, United States). The sampling rate was at 1,500 Hz. The bipolar self-adhesive Ag/AgCl surface electrodes, with a 20 mm inter-electrode distance, were placed in parallel to muscle fiber orientation after alcohol was used to cleanse each participant’s skin to decrease skin impedance. EMG signals were collected from eight trunk muscles bilaterally: EO, ES, MF, and rectus abdominis (RA) (Table 1).

**TABLE 1:** Placement of surface electromyography electrodes and measurement of the maximum voluntary contraction of trunk muscles.

Muscle	Placement of electrodes	Maximum voluntary contraction test
Rectus abdominis	3 cm lateral to the umbilicus	Participants were in the supine position with knees bent and feet flat and were asked to flex against manual resistance at the shoulders
External oblique	15 cm lateral to the umbilicus	Participants were in the supine position with knees bent and feet flat and were asked to laterally bend and axially twist against manual resistance at the shoulders
Erector spinae	3 cm lateral to the L3 spinous process	Participants were in the prone position. Then, trunk extension was performed against manual resistance at the shoulders
Lumbar multifidus	3 cm lateral to the L5 spinous process	Test was the same as the test of the erector spinae muscle

To normalize the EMG data, the participants were instructed to perform a maximal voluntary contraction (MVC) test according to SENIAM recommendations (Hermens et al., 2000), and more details are given in Table 1. A total of two repetitions of the 5-s isometric MVC test were performed for each target muscle against manual resistance, with a 2-min rest between trials; the order of the test was randomly assigned. After the measurement of MVC, all participants randomly performed the HKK and HFK movements according to the procedure.

The raw EMG data were measured using automated programs written in MATLAB version R2018a (MathWorks, Inc., Natick, United States). During postprocessing, the first 3 s and the last 2 s data were discarded. The discarded EMG signal was quantified by digital full-wave rectifying and band-pass filtering (at 20–500 Hz) and smoothed with a root-mean-square (RMS) algorithm with a 100-millisecond moving window. The average EMG signal amplitude of each muscle during the MVC trials was regarded as representing 100% muscle activity, and then the average EMG data of each exercise were normalized to a percentage of the average of the MVC.

### Kinematics data acquisition and processing

The Formetric 4D analysis system (DIERS International GmbH, Schlangenbad, Germany) was used to measure vertebral rotation, which is a radiation-free and contact-free method based on raster stereography (Drerup and Hierholzer, 1996). The process takes only seconds, unlike traditional spinal motion laboratory system measurement methods that require multiple markers to describe the limited number of spine segments’ motion and need complex post-data processing (Papi et al., 2017; Ryan and Bruno, 2017).

Parallel light lines are projected onto the surface of the back and detected using a digital camera. From the distortion of the raster lines and with the help of a personal computer, the three-dimensional model of the spine was reconstructed. By a mathematical shape analysis, the frontal, the sagittal, and the transversal profile can be determined (Drerup and Hierholzer, 1994). The reliability and validity of the Formetric raster stereography device had been proved in previous studies (Betsch et al., 2011; Schroeder et al., 2015).

**FIGURE 2:**
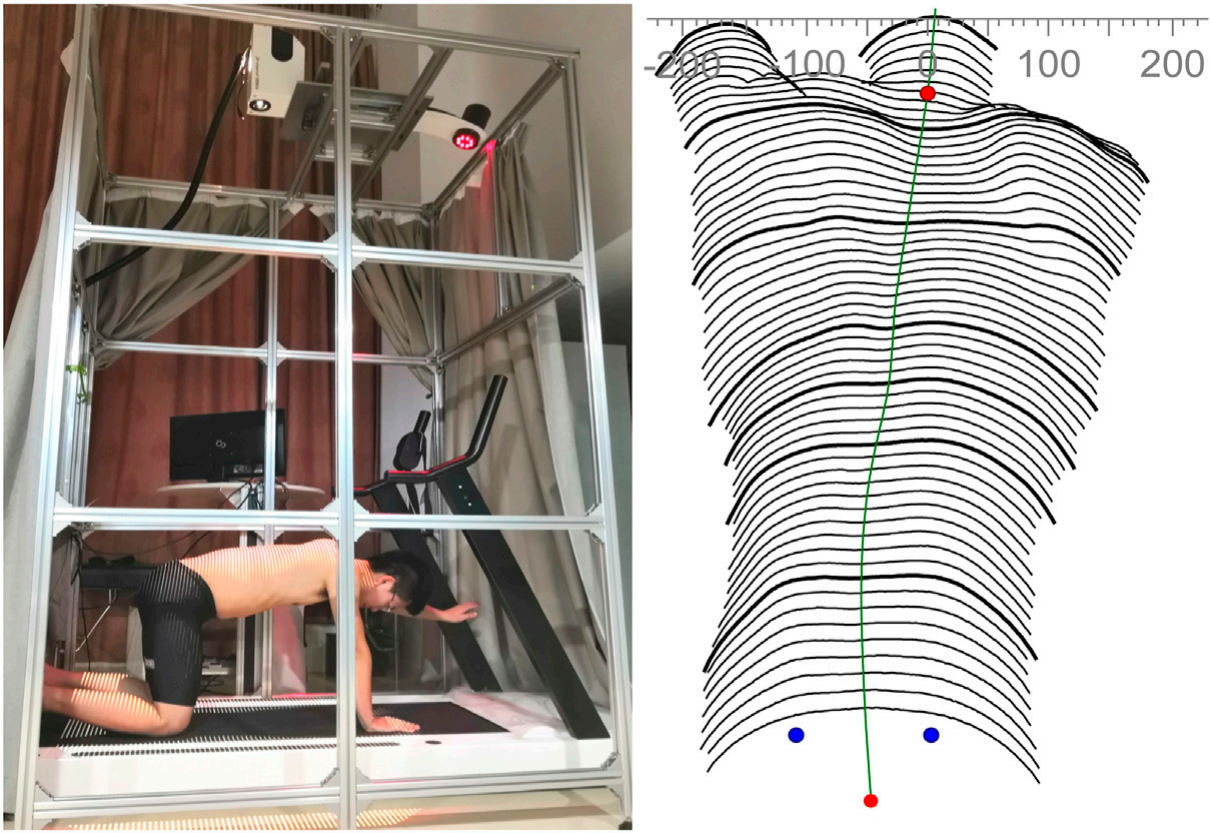
Modified Formetric 4D device (left panel) and computerized surface topography map of the participant’s back (right panel). The distance between the camera and the participant’s back is about 2 m.

All parameters were measured using the function tool four-dimensional average (4D average). The duration of image acquisition was set at 6 s, and the frequency was set at 2 Hz. Moreover, the original design of equipment was to measure the spine in the standing position; we modified the equipment to fulfill the experiments. As shown in Figure 2, the distance between the camera and the participant’s back was about 2 m, and the height of the camera could be fine-tuned to suit different participants. More details of the preparation are referred to Gipsman et al. (2014).

The collected data were processed by software automatically. The averages of the vertebral rotations were calculated, and the negative value and the positive value meant that the vertebra rotates to the left and right in the data set, respectively.

### Statistical analysis

All the statistical analyses were performed using SPSS statistical software (version 20.0; Inc., Chicago, IL, United States), and statistical significance was accepted at *p* < 0.05. The paired *t*-test was utilized to compare them between the two positions with the same limb lifted. The one-way ANOVA test with the Bonferroni correction was utilized to compare the muscle activity and vertebral rotation between five positions in both HKK and HFK, respectively.

## Results

### Muscle activity

**FIGURE 3:**
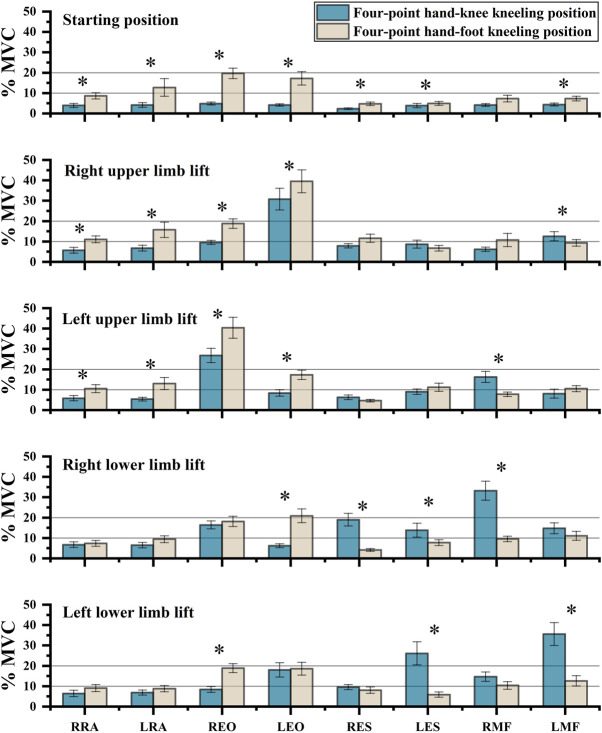
Comparison of sEMG values between the four-point hand−knee and hand−foot kneeling positions with the same limb lifted. RRA, right rectus abdominis; LRA, left rectus abdominis; REO, right external oblique; LEO, left external oblique; RES, right erector spinae; LES, left erector spinae; RMF, right lumbar multifidus; LMF, left lumbar multifidus. *: statistically significant, *p* < 0.05.

The *t*-test showed the activation of the same muscle between HKK and HFK with the same limb lifted was statistically different (Figure 3). The activation of both sides of RA (*p* < 0.05), EO (*p* < 0.001), ES (*p* < 0.05), and left MF (*p* = 0.009) in HFK were higher than that in HKK in the starting position. The activation of both sides of RA and EO in HFK was higher than that in HKK when the upper limb was lifted (*p* < 0.05), while the activation of contralateral MF was higher in HKK than in HFK when the upper limb was lifted (*p* < 0.05). The activation of ipsilateral MF and ES in HKK was higher than that in HFK when the lower limb was lifted (*p* < 0.05), while the activation of EO muscles was higher in HFK than in HKK when the contralateral lower limb was lifted (*p* < 0.05).

**FIGURE 4:**
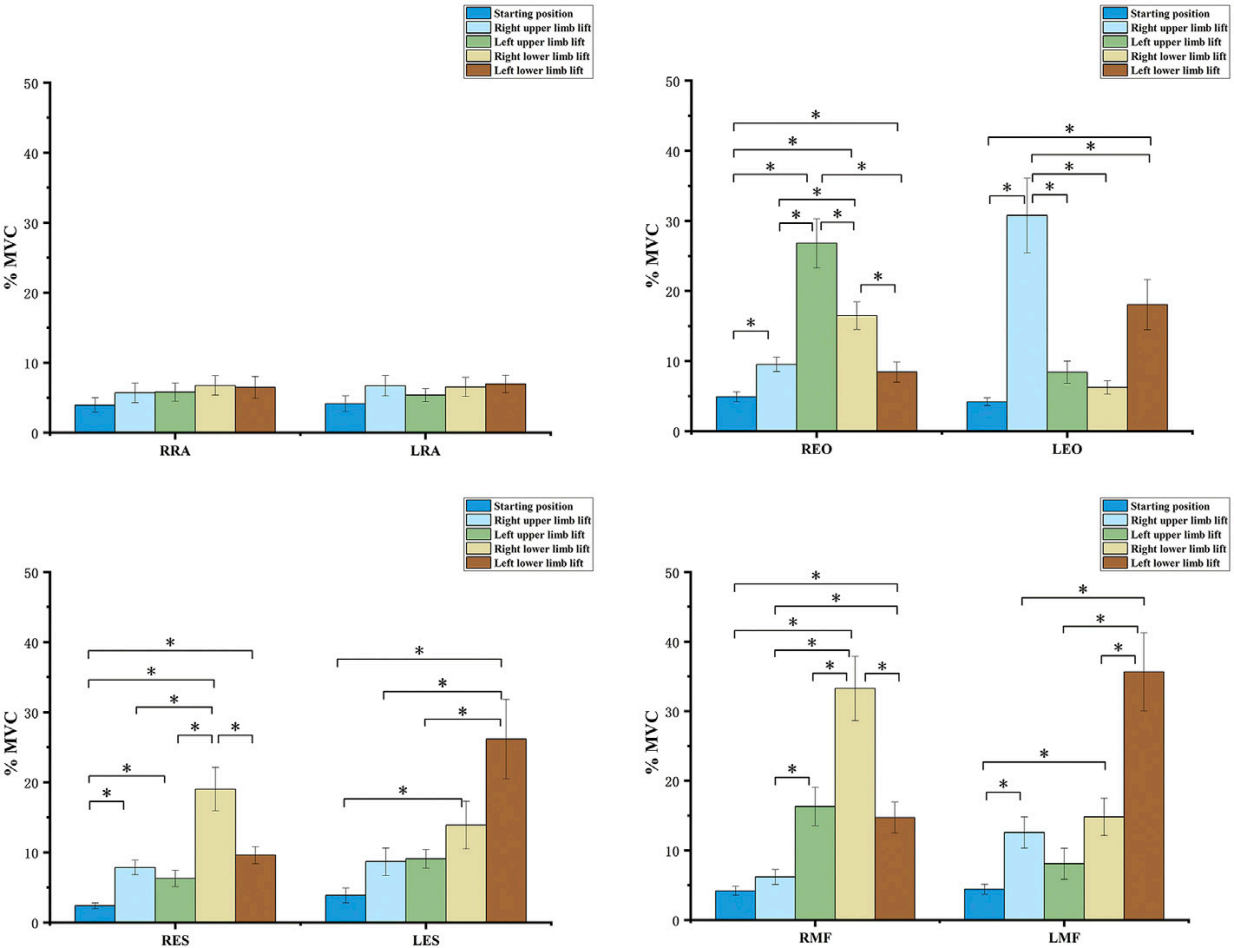
sEMG values and results of one-way ANOVA during the four-point hand−knee kneeling positions, respectively. The negative value meant the vertebra rotates to the left, and the positive value meant the vertebra rotates to the right; RRA, right rectus abdominis; LRA, left rectus abdominis; REO, right external oblique; LEO, left external oblique; RES, right erector spinae; LES, left erector spinae; RMF, right lumbar multifidus; LMF, left lumbar multifidus. *: statistically significant, *p* < 0.05.

**FIGURE 5:**
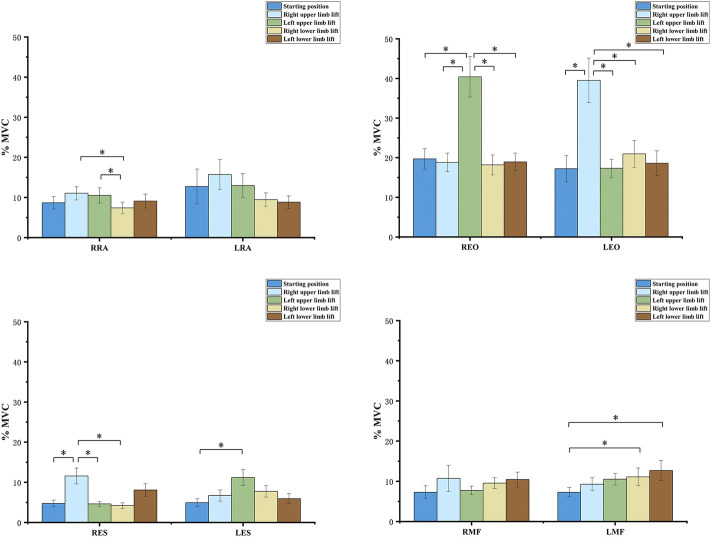
sEMG values and results of one-way ANOVA during the four-point hand−foot kneeling positions, respectively. The negative value meant the vertebra rotates to the left, and the positive value meant the vertebra rotates to the right. RRA, right rectus abdominis; LRA, left rectus abdominis; REO, right external oblique; LEO, left external oblique; RES, right erector spinae; LES, left erector spinae; RMF, right lumbar multifidus; LMF, left lumbar multifidus. *: statistically significant, *p* < 0.05.

The one-way ANOVA test for comparing the five exercises in the HKK position showed significant differences in the muscle activation of most trunk muscles (*p* < 0.001), except the left RA (*p* = 0.076) (Figure 4). During exercises in the HFK position, statistically significant differences were found in the activation of the muscles (*p* < 0.05), except for the left RA (*p* = 0.161) and right MF (*p* = 0.263) (Figure 5).

### Vertebral rotation

**FIGURE 6:**
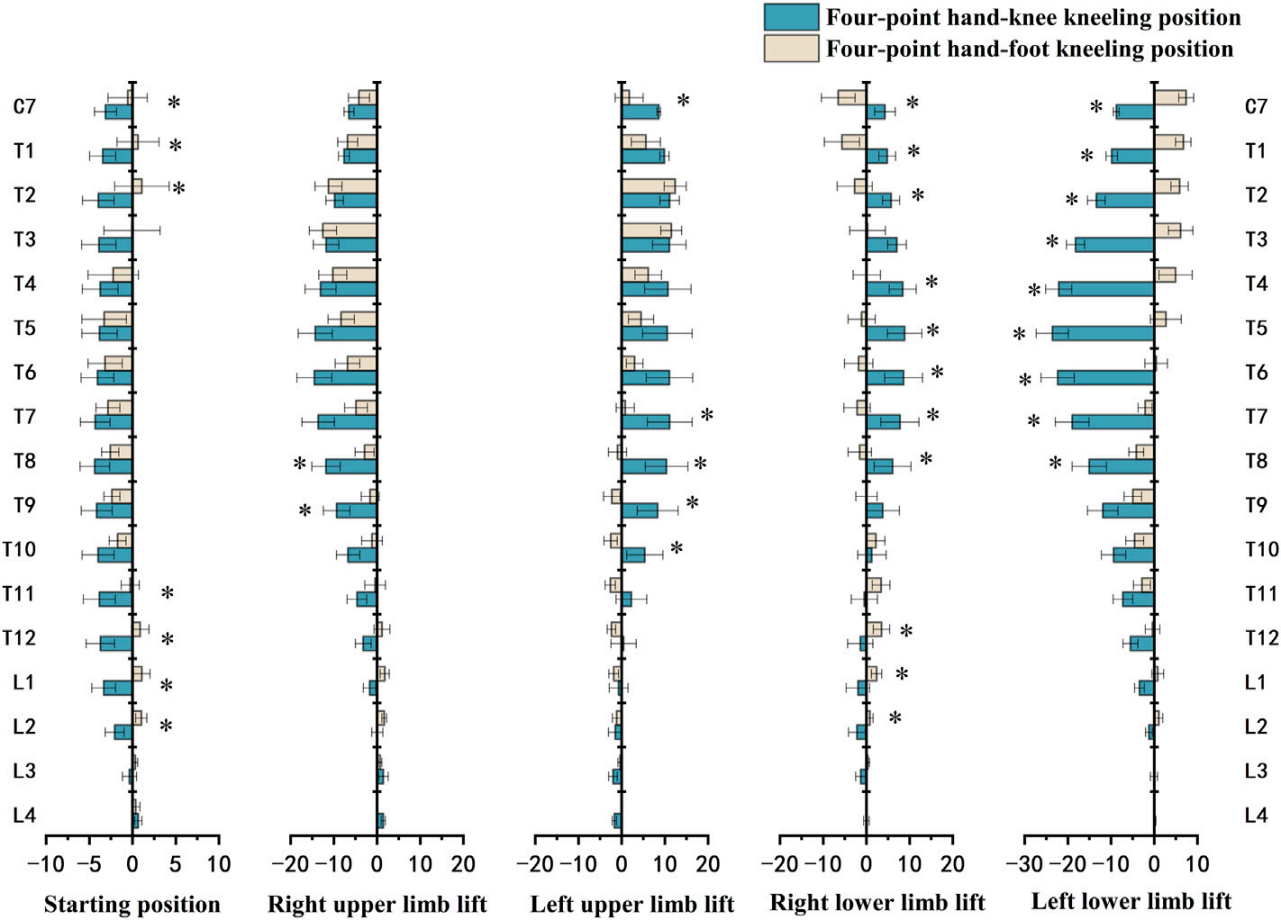
Comparison of vertebral rotation (°) between the four-point hand−knee and hand−foot kneeling positions. The negative value meant the vertebra rotates to the left; the positive value meant the vertebra rotates to the right. *: statistically significant, *p* < 0.05.

The vertebral rotation between two positions with the same limb lift was compared (Figure 6). The results showed that the difference was mainly at T8 and above (*p* < 0.05) when the lower limb was lifted, while the difference existed at T7–T10 during exercises with the upper limb lifted (*p* < 0.05). Moreover, the exercises in the HKK position had a greater vertebral rotation angle than those in the HFK position, unless the right upper limb was lifted, although the rotation might be in a different direction.

**FIGURE 7:**
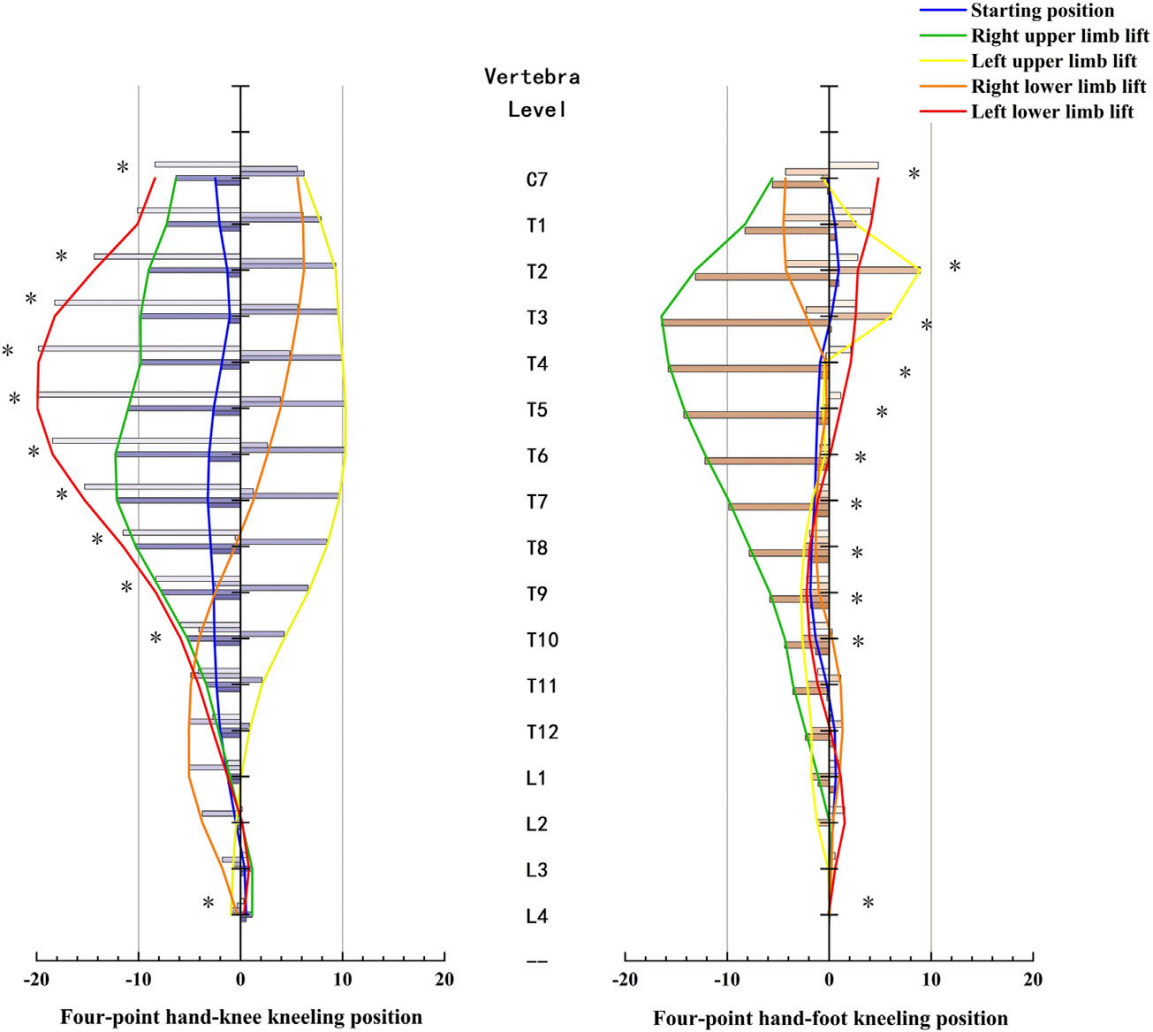
Vertebra rotation and results of one-way ANOVA during the four-point hand−knee kneeling positions and four-point hand−foot kneeling positions, respectively. *: statistically significant, *p < 0.05.*

The rotation angle of each vertebra from C7 to L4 for each exercise in the HKK and HFK positions is displayed in Figure 7. In HKK, the results showed that the thoracic vertebral rotation (from T1 to T7) was significantly larger when the left lower limb was lifted than in the starting position (*p* < 0.05), and the thoracic vertebra (from T1 to T8) showed the opposite rotation direction when the left upper limb was lifted and the left lower limb was lifted. However, there were no significant differences in the lumbar spinal rotation (from L1 to L3) between positions with different limbs lifted in both HKK and HFK.

## Discussion

The purpose of this study was to quantify muscle activation and spine kinematics during movements in HKK and HFK to compare the differences between these two quadruped positions in training core muscle function and spinal mobility. The muscle activity data in HKK were consistent with some earlier results (Beith et al., 2001; Stevens et al., 2007b; Pirouzi et al., 2013). Furthermore, our study provided comprehensive comparisons of bilateral muscle activation in two four-point kneeling positions with specific limb lift.

According to the classification of muscle activation, the relative EMG levels (%MVC) were classified into high (>20% MVC), moderate (10%–20% MVC), and low (<10% MVC) muscle activity (Stevens et al., 2007b). In the starting position, all muscle activation was low (<10% MVC) during HKK. Only one study showed that the activation of all muscles was less than 10% in the HKK position (Stevens et al., 2007a). This result was in line with our study. The activation of the RA, EO, ES, and left MF was higher during HFK than during HKK in the starting position and with the upper limb lifted. Previous studies have indicated that abdominal trunk muscle strength decreases with chronic low back pain (Cho et al., 2014; Cruz-Díaz et al., 2017). Moreover, Kato et al. (2019) found that abdominal muscle training could effectively improve transversus abdominis muscle activation in patients with chronic low back pain and was associated with pain and function improvement. HFK in the starting position and with the left limb lifted could provide more training for strengthening abdominal muscles and might be used for people with chronic low back pain caused by abdominal muscle weakness.

The ES and MF had higher activity during HKK than during HFK with the ipsilateral lower limb lifted. A previous study showed that HKK with the right lower limb lifted had higher activity of the right EO and MF muscles and lower activity of the RA, which was consistent with our results (Stevens et al., 2007b). The ES and MF were thought to play an important role in the active stabilization and movement of the spine (Sadeghi et al., 2019; Kouwijzer et al., 2020). Previous studies showed that people with low back pain were found to have smaller multifidus muscles with a significant amount of intramuscular fat infiltration (Seyedhoseinpoor et al., 2021). Considering the high activation of ES and MF (>20% MVC) during HKK with the lower limb lifted, it could be used to strengthen ES and MF in particular.

The findings of the present study also revealed that the muscle activation was closely related to the uplifted limb, and the relationship was affected by whether the knee touched the ground. Analyzing the moderate and high activated muscles during exercises, we could conclude the following: the EO and MF muscles had a higher muscle activity during HKK with the contralateral upper limb or the ipsilateral lower limb lifted, and the EO muscle activation was higher during HFK with the contralateral upper limb lifted. HKK with the lower limb lifted had a highly activated ipsilateral ES muscle. Based on these results, we suggested the specific variant could strengthen specific muscles.

The results showed that the rotation angle of the thoracic spine in HKK with the lower limb lifted was significantly larger than that in HFK, and the results also showed that the rotation angles of T7–T10 in HKK with the upper limb lifted were significantly larger than those in HFK. A previous study focused on spine motion during axial rotation activities also showed the greatest degree of axial rotation of the upper thoracic spine in comparison with the other spinal segments (Sung et al., 2012). Yang et al. (2015) pointed out that motion reduction of the thoracic segments caused excessive movements of the lumbar spine *via* compensatory mechanisms. The abnormal movements of the lumbar spine led to the instability that develops in the facet joints, which eventually causes pain in the lower back (Panjabi, 1992). However, the results showed that little rotation angles of the lumbar were observed in HKK and HFK because the high activation of the trunk muscle led to intervertebral joint stiffness, which restricted the axial rotation (Lee et al., 2006; Graham and Brown, 2014; Inoue et al., 2020).

The vertebral rotation was also closely related to the uplifted limb in different positions. For example, in HKK and HFK, the thoracic spine rotated oppositely to the side of the uplifted upper limb. However, the direction of the thoracic rotation was on the same side of the raised lower limb in HKK, while that in HFK was on the opposite. Recently, some studies also showed that thoracic mobilization exercises had positive outcomes in relieving pain and functional disability in patients with chronic low back pain (Divya et al., 2020; Kostadinović et al., 2020). In our research, HKK with limbs lifted increased thoracic mobilization and reduced lumbar hypermobility, which might be used in low back pain management.

In the starting position, the rotation angles of T11–L2 during HKK were significantly larger than those during HKF. However, there were no significant differences in most thoracic rotation angles between HKK and HFK, and relatively little rotation angles were shown in both HKK and HFK. Because the body weight is supported by two knees (feet) and two hands in the starting position, which is a stable posture, the neutral spine position could be easily achieved (Pirouzi et al., 2013). Also, previous studies showed that trunk muscles activated less when in the starting position than when the limbs were lifted. (Stevens et al., 2007b; Chanthapetch et al., 2009). This result was in line with our findings in muscle activation. Thus, it was a suitable choice for people at the beginning of rehabilitation programs for low back pain or spinal disorders due to the little physical demands needed.

### Limitations

There are some limitations to this study. First, all the participants recruited were young healthy subjects. Further studies need to take people with low back pain or the elderly into consideration. Second, the beneficial effects need to be identified by long-term intervention based on two kinds of four-point kneeling positions.

## Conclusion

The exercises in both four-point hand−knee kneeling positions could train core muscle and spinal mobility and could be suitable choices for people with low back pain or spinal disorders. The exercises in the HFK (deer play) position could provide more training for strengthening abdominal muscles, while the exercises in the HKK position with the lower limber lifted could be recommended for strengthening lumbar muscles. HKK and its variants were presumably suitable for increasing spine mobility. The findings in this study can be used to help physiotherapists, fitness coaches, and others to select specific exercises and develop individualized training programs.

## Data Availability

The raw data supporting the conclusion of this article will be made available by the authors, without undue reservation.
